# Composition and variability of core phyllosphere fungal mycobiota on field-grown broccoli

**DOI:** 10.1186/s40793-023-00474-0

**Published:** 2023-03-01

**Authors:** Min-Soo Kim, Eun-Jin Park

**Affiliations:** 1 Department of Microbiology and Molecular Biology, Chungnam National University, 99 Daehak-ro, Yuseon-gu, Daejeon, 34134 Republic of Korea; 2 Department of Food Bioengineering, Jeju National University, 102 Jejudaehak-ro, Jeju, 63243 Republic of Korea

**Keywords:** Phyllosphere mycobiota, Preharvest microbiome, Broccoli, Microbial diversity, Metagenomics

## Abstract

**Background:**

Fresh vegetables harbor an assemblage of different microorganisms on their surfaces. The phyllosphere microbiota is important for maintaining plant health and managing crop quality before and after harvest. However, the diversity and ecology of fungal communities are largely unexplored in fresh vegetables. This study investigated the phyllosphere mycobiota of field-grown broccoli florets (n = 66) collected from 22 farms across four regions in Korea, using culturing, amplicon sequencing of the internal transcribed spacer region, and microbial network analysis.

**Results:**

Microbial network analysis identified core genera (*Purpureocillium*, *Filobasidium*, *Cystofilobasidium*, *Papiliotrema*, *Aureobasidium*, and unclassified genera of Capnodiales) specific to the broccoli phyllosphere. The composition and network complexity of core and unique populations varied among farming regions, and was associated with local agro-meteorological conditions. The complexity of microbial associations was higher in mature communities than in immature communities, but complexity was lost upon development of plant pathogenic disease. Broccoli mycobiota were classified according to the dominance of *Purpurecillium*. While *Purpurecillium*-type microbiota were prevalent in normal samples, *Filobasidium*-type microbiota were frequently observed in immature, damaged, or postharvest samples.

**Conclusions:**

Together, fungal communities were important components of phyllosphere microbiota on fresh vegetables, and have substantial potential for exploitation to enhance and stabilize plant health and growth.

**Supplementary Information:**

The online version contains supplementary material available at 10.1186/s40793-023-00474-0.

## Background

Plants harbor diverse and abundant microorganisms on their surfaces, and have co-evolved mutually beneficial interactions with their microbial communities [1]. Host–microbe interactions play an important role in stabilizing plant growth and health [2], and these interactions have substantial potential to be utilized for enhancement of crop production in horticulture and agriculture [3, 4]. Fresh vegetables harbor different types of microbial populations on their leaf surfaces during growth [5, 6], and the phyllosphere microbiota vary according to host development [7], farming region [8, 9], agricultural practice [10, 11], and climate [9, 12]. Previous research has widened our knowledge of bacterial communities on fresh vegetables [5, 13]; however, fungal communities remain largely unexplored.

Food loss in fresh produce represents nearly half of all food wastage [14], with major losses occurring during postharvest storage [15]. A range of fungi is known to adversely affect food spoilage, and food losses due to fungal spoilage account for up to 10% of all losses [16]. Hence, studies of fungal microbiota have focused primarily on postharvest fungi, particularly plant pathogens [17]. Importantly, microbial communities that develop during plant growth persist long after harvest [11], and the initial composition and diversity of preharvest microbiota have an important influence on the communities that develop during postharvest storage [18]. Furthermore, preharvest microbiota have also been shown to contribute to food spoilage in harvested crops [19], via antagonistic or agonistic interactions between commensal and pathogenic community members [3, 20]. Such interspecies interactions can be exploited to suppress the growth of potential plant pathogens and improve crop quality by treatment with biocontrol strains in the field and during postharvest storage [21]. Understanding the diversity and ecology of the phyllosphere mycobiota at the preharvest stage provides a foundation for strategies to maintain the quality and safety of fresh vegetables in the food industry [22, 23].

The ecology of plant-associated microbiota reveals strong dependencies on host and environmental factors, resulting in high complexity and variability of plant microbiota [24]. Nonetheless, plants harbor a subset of core taxa that are persistently and directly associated with a variety of different conditions [25]. The pervasive nature of core microbiota provides an expectation that they play important roles in the regulation of host physiology and immunity [26], and can be viewed as microbial resources for the development of biological control agents in economically important crops, including fresh vegetables [21]. Constructing microbial associations can facilitate the prediction of inferred interactions between co-habiting members in a community and contribute to disentangling the complexity of microbial communities under a broad array of host and environmental conditions [27]. It is also considered that diverse microbial communities likely have a high probability of containing microorganisms potentially antagonistic to plant pathogens, or agonistic to host plants as a result of co-evolution [28]. Therefore, identifying co-occurrence relationships between community members provides an opportunity to elaborate core keystone species and to predict their ecological functions with the aim of developing biocontrol strategies.

Here, 66 broccoli florets were collected at the point of harvest from 22 local farms in four regions of Jeju Island, Korea, and used to characterize the diversity and ecology of the phyllosphere mycobiota on field-grown broccoli. Broccoli (*Brassica oleracea* var. *italic*) is a valuable *Brassica* vegetable with global popularity, and Jeju Island is a major producer of broccoli in Korea with up to 80% of market share [29, 30]. Comparison of fungal communities identified the variability of fungal community composition according to farming region, disease occurrence, and host development stage, and the construction of microbial association networks allowed variation in interspecies associations of fungal communities under different conditions to be understood, revealing the core taxa of phyllosphere mycobiota on field-grown broccoli.

## Methods

### Sample collection and preparation

All procedures for sample collection and preparation were described precisely [8, 31]. Briefly, 66 florets of broccoli were collected from 22 farms distributed in four regions of Jeju Island (six farms in Daejeong [A], five farms in Hallim [B], five farms in Jocheon [C], and six farms in Seongsan [D]) during harvest season from November 2014 to February 2015 (Additional file 1: Fig. S1). The florets were collected from immature plants grown for 8–9 weeks after seedlings planted in the soil (weight, 80–120 g) at one of each region, and mature plants grown for 15–16 weeks (250–400 g) at the remaining farms of each region. The Agricultural Research and Extension Services of Jeju reported that broccoli plants at five farms in region C were physically damaged by black rot and downy mildew in late 2014 before sampling. Retail samples (n = 40) were collected from ten local grocery stores of Jeju Island from November 2014 to January 2015, and November 2015. Three sections (approximately 10 g per section) were taken using a sterile knife and pooled to be 30 g per sample. Floret samples were mixed with 120 ml of 0.1% buffered peptone water (Difco, Becton Dickinson, Sparks, MD, USA), and sonicated for 10 min at maximum power (Powersonic420, Hwashin Technology, Republic of Korea). Microbial pellets were obtained by centrifugation at 16,000 × *g* at 4 °C. Agro-meteorological data (temperature, relative humidity, precipitation, insolation, wind speed, soil temperature and soil moisture) at the sampling date were provided by the Jeju Agricultural Research and Extension Services (https://ipm.agri.jeju.kr/develope/weather/weather0102.php). The weather observation stations are located within 3.4 ± 1.6 km (4.4 ± 0.6 km for region A, 3.2 ± 0.4 km for region B, 0.9 ± 0.6 km for region C, and 4.6 ± 1.0 km for region D) at the shortest direct distance from the local farms (Additional file 2: Table S1).

### DNA extraction and internal transcribed spacer sequencing

Metagenomic DNAs were extracted from microbial pellets using a PowerSoil DNA Isolation Kit (MO-BIO Laboratories Inc., Carlsbad, CA, USA). The internal transcribed spacer 2 (ITS2) region was amplified using ITS86F and ITS4 primers with Illumina sequencing overhang adapters [32]. PCR was performed, as described previously [33]. DNA extraction and PCR amplification were unsuccessful for 15 samples (A5, A9, A13, A17, A18, B27, B29, B32, B33, C34, C43, C48, D54, D55, and D58) which were excluded from sequencing. Five PCR replicates were purified using a QIAquick PCR Purification Kit (Qiagen, Valencia, CA, USA), and sequenced using Illumina MiSeq sequencing (2 × 300 bp) (Illumina, San Diego, CA, USA).

### Internal transcribed spacer sequence analysis

Illumina sequences were analyzed using QIIME2 (version 2018.11) [34], as described previously [33]. Briefly, primer sequences were trimmed, and conserved regions flanking the ITS2 region were removed using ITSxpress [35, 36]. Divisive Amplicon Denoising Algorithm 2 (DADA2) [37] was used to identify amplicon sequence variants (ASVs). Samples were even-depth rarefied to 15,000 sequences, and ASVs with < 6 sequences were removed. Fungal taxonomies were defined using the UNITE QIIME release for Fungi (clustering at 99% similarity, release 2020-02-04) [38]. The number of ASVs, Shannon index, and Pielou's evenness were estimated. Beta-diversity was determined based on Bray–Curtis dissimilarity and Jaccard distance. Global clustering was performed based on Jensen-Shannon divergence using partitioning around medoids clustering algorithm [39, 40]. The optimal number of clusters was selected with the highest value of Calinski-Harabasz index.

### 16S ribosomal RNA gene sequence analysis

The 454 pyrosequencing data of 16S rRNA amplicon in our previous study [8] were reassessed using QIIME2. Primer sequences were trimmed away, and quality-filtering, error correction, and removal of chimeric sequences were performed using DADA2. The taxonomy of ASVs was defined using the de-replicated sequences (99% similarity) of Greengenes database (2013-08 release). Mitochondria or chloroplast ASVs were filtered out. The sequencing depth was rarefied to 500 sequences, and ASVs with < 2 sequences were excluded.

### Co-occurrence network analysis

Spearman correlations were calculated using CCREPE [41], and positive and negative correlations with statistical significance (correlation coefficient > 0.4, *P*-value < 0.01) were used. A microbial association network was visualized using Compound Spring Embedder Layout in Cytoscape 3.8.2, with global graph properties including total nodes, total edges, average neighbors, and cluster coefficient. Fungal data were further rarefied to 500 sequences, and retained ASVs with > 2 sequences for the construction of fungal and bacterial co-association networks.

### Enumeration of fungal and bacterial cells

Total numbers of viable fungal and bacterial cells were determined by counting colony-forming units (CFUs). Sample mixture was serially diluted and spread on tryptic soy agar (Difco, Becton Dickinson) for total bacteria, and potato dextrose agar (Difco, Becton Dickinson) for total fungi. Plates for fungi and bacteria were incubated at 30 °C for 72 h and at 37 °C for 48 h, respectively.

### Statistical analysis

*P*-value of < 0.05 were considered significant. Two-tailed Mann–Whitney U, Kruskal–Wallis tests with Dunn's post-hoc, and one-way ANOVA with Tukey’s post-hoc, χ^2^-test, and Fisher’s exact test were performed using GraphPad Prism version 5.0 for Windows (GraphPad Software, SD, USA). Principal coordinate analysis (PCoA) was performed using R package ‘vegan’ and ‘ade4’ [42]. Statistical significance for distance matrices was assessed using the ‘adonis’ function of ‘vegan’ package, and significance for pairwise comparisons was assessed using the ‘pairwise.adonis’ function of ‘pairwiseAdonis’ package. A z-score was used for standardization of agro-meteorological variables. Spearman correlation between distance matrices was calculated using the ‘mantel’ function of ‘vegan’ package. Procrustes analysis between ordinations was performed using the ‘protest’ of ‘vegan’ package. Distance-based redundancy analysis (db-RDA) was performed using the ‘capscale’ of ‘vegan’ package. The best-fit model was calculated using forward selection of ordiR2step in ‘vegan’ package. The abundance of ASVs was correlated with the axes of constrained ordination using the ‘add.spec.scores’ of ‘BiodiversityR’ package, and statistical significance was achieved using the ‘envfit’ of ‘vegan’. Discriminant analysis was identified using LEfSe analysis [43].

## Results

### Taxonomic uniqueness of fungal populations in the broccoli phyllosphere

**Fig. 1:**
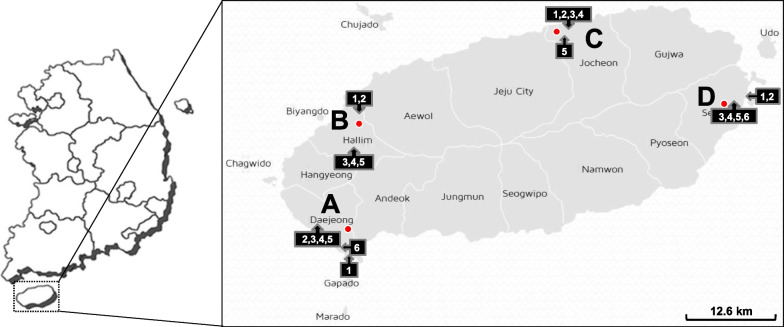
The sample collection sites of broccoli florets, Jeju island, Korea (downloaded from http://www.visitjeju.net/en/index.jto). Red circles indicate weather observation stations that are the nearest to the sampling sites

**Fig. 2:**
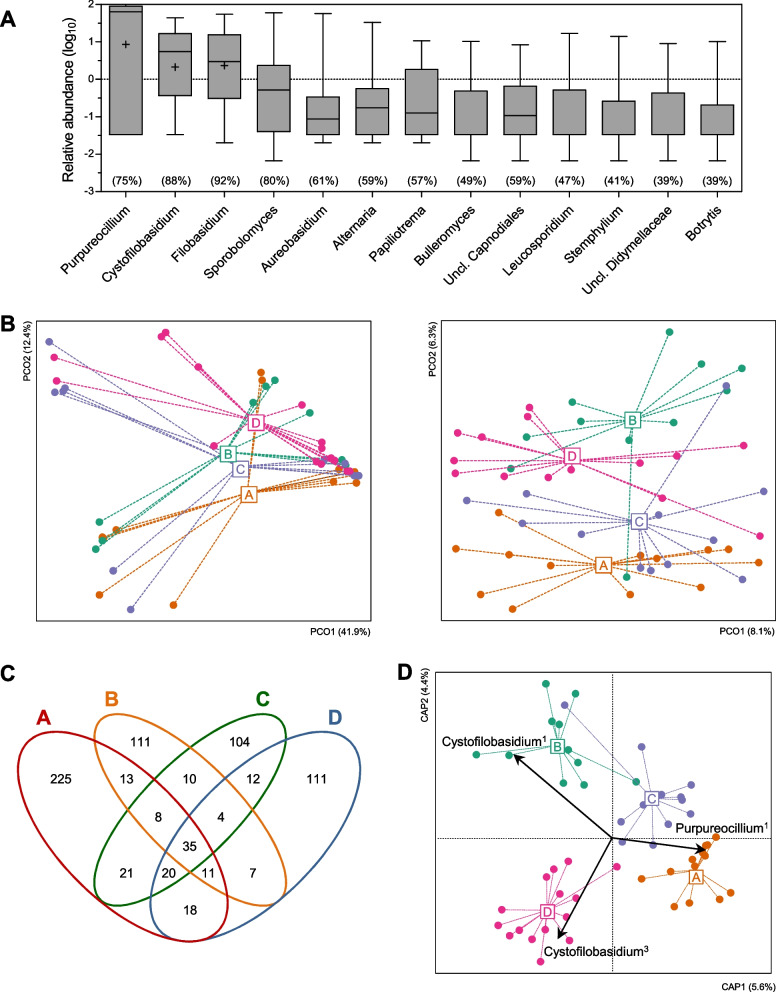
Fungal community composition in field-grown broccoli florets. **A** The genus-level core members of the broccoli mycobiota. Abundant (> 0.4% average relative abundance) and prevalent (> 39% samples) fungal genera are shown using a rank abundance plot. The prevalence of fungal genera indicates in parentheses. Box and whisker plots are shown to min and max. **B** The fungal communities of four farming regions were compared using Principal coordinates Analysis with Bray–Curtis dissimilarity (left panel) and Jaccard distance (right panel). **C** Venn diagram showing shared ASVs among the farming regions. **D** Farming region-discriminant ASVs were predicted by correlation with the constrained axis of distance-based redundancy analysis with Jaccard distance for farming region (*r* > 0.4, *P* < 0.01)

In total, 66 florets of field-grown broccoli were collected from 22 farms located on Jeju Island over two years (Fig. 1) [8]. On average (± s.d.), 86,992 ± 52,077 high-quality reads of the ITS2 region were obtained per sample (n = 51), and ASVs representing species-level biological variants were defined. Samples were rarefied to minimize the effect of variable sequencing depth, and ASVs with very low abundance were filtered out (see Methods). Finally, a total of 710 ASVs were obtained, with 34 ± 24 ASVs produced per sample (Additional file 2: Table S1). Most (89.3%) of the ASVs belonged to 256 genera of the *Ascomycota* (66.7 ± 31.4%) and *Basidiomycota* (32.6 ± 31.0%) phyla. Thirteen genera conformed to the following two conditions and were regarded to as core genera: 1) observed at > 0.4% average relative abundance, and 2) found in ≥ 39% of samples (Fig. 2A). The most abundant core genera (> 1%) were *Purpureocillium* (52.0 ± 38.8%), *Cystofilobasidium* (10.3 ± 12.6%), *Filobasidium* (9.8 ± 13.4%), *Sporobolomyces* (3.9 ± 10.1%), *Aureobasidium* (1.5 ± 7.9%), *Alternaria* (1.3 ± 4.7%), and *Papiliotrema* (1.2 ± 2.0%). These core genera have not been previously been observed at high frequency in fresh produce [3, 44–51], suggesting that field-grown broccoli harbors unique fungal populations on their surfaces.

### Regional variation in the fungal communities of the broccoli phyllosphere

In total, 710 ASVs were used to compare the composition of fungal communities on field-grown broccoli based on Bray–Curtis dissimilarity for ASV abundance and Jaccard distance for ASV occurrence. The fungal communities differed significantly among farming regions with respect to ASV occurrence (Adonis, *p* = 0.001), but not to ASV abundance (*p* = 0.209) (Fig. 2B). Regional differences were further assessed using pairwise comparisons, and post-hoc analysis revealed that significant differences were found for all pairs of fungal communities for Jaccard distance (*p* < 0.05) (Additional file 1: Fig. S2A). These results indicate that fungal communities of field-grown broccoli differ among farming regions.

Fungal community members were stratified to identify influential ASVs driving regional variation of the broccoli mycobiota. Interestingly, the majority of fungal ASVs were allocated exclusively to one of the regions (Fig. 2C). Specifically, 35 ASVs (4.9%) regarded as “core” ASVs were shared among all farming regions, whereas 551 ASVs (77.6%) regarded as “unique” were observed only in single regions (Fig. 2B). Relative abundance of core and unique ASVs were examined to determine their dominance or rarity in their communities. The core and unique ASVs showed total abundance averages (± s.e.m.) of 2.2 ± 0.1% and 0.2 ± 0.1%, respectively, and the core ASVs were significantly more abundant than the unique ASVs (*p* < 0.0001) (Additional file 1: Fig. S2B). These data reveal that rare populations are a main driver for regional variation in the fungal communities.

Influential ASVs contributing to regional variation of the fungal communities were also identified from abundant ASVs. Fungal ASVs (> 0.1% average abundance) were identified that significantly correlated with the first two axes of db-RDA constrained to farming region. Three ASVs belonging to the core genera had significant correlations with the first or second axis of the constrained db-RDA for Jaccard distance (*r* > 0.4, *p* < 0.05) (Fig. 2D). The *Purpureocillium*^1^ ASV was frequently observed in regions A and C (Additional file 1: Fig. S2C). One *Cystofilobasidium* ASV was abundant in region B, and the other *Cystofilobasidium* ASV was abundant in regions A and D (Additional file 1: Fig. S2C). Thus, dominant core populations also contribute to regional variation in the fungal communities.

### Agro-meteorology is associated with regional variation in the fungal communities

**Fig. 3:**
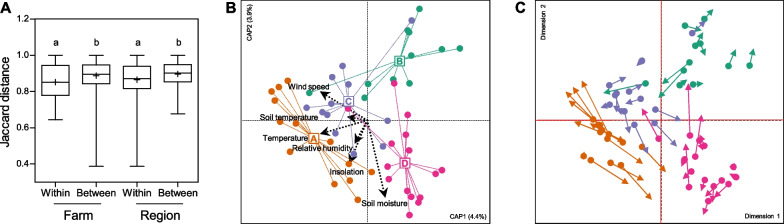
The influence of agro-meteorology on regional variation of fungal communities. **A** Comparison of Jaccard distance within and between farms and regions. Statistical significance was determined by one-way ANOVA with Tukey’s multiple comparison test. **B** The best-fit model of fungal communities for six agrometeorological factors in distance-based redundancy analysis with Jaccard distance. **C** The Procrustes plot of distance-based redundancy analysis plots based on Jaccard distance was shown for community-wide association between agro-meteorology and farming region

Samples were collected from multiple farms within the same region, and fungal communities can be presumed to be influenced by farm-based local environments. Dissimilarities between samples collected from the same farm or region were significantly smaller than those from different farms or regions (*p* < 0.05), but dissimilarities between samples collected from different farms were not smaller than those from different regions (Fig. 3A), indicating that region-based conditions may prevail over farm-based conditions with respect to fungal community composition.

Agricultural meteorology is a key contributory factor influencing diversity of plant-associated microbiota [24], and we hypothesized that fungal community composition was affected by the climate conditions of each region. Agro-meteorological parameters (temperature, relative humidity, precipitation, insolation, wind speed, soil temperature, and soil moisture) were collected at the sampling time from weather observation stations at an average distance of 3.4 ± 1.6 km from the sampling locations (Additional file 2: Table S1). First, the Euclidean distance matrix of the agro-meteorological parameters was compared with the Jaccard distance matrix of the fungal communities using a Mantel test, and significant Mantel statistics were observed for the matrix comparisons (*r* = 0.2329, *p* = 0.006). This provides evidence that agro-meteorological conditions are associated with regional variation in the fungal communities.

Next, the best-fit model of Jaccard distance-based db-RDA plot constrained to optimized number of agro-meteorological parameters was developed using redundancy analysis with forward selection. The model fitted by six parameters (soil moisture, temperature, soil temperature, relative humidity, wind speed, and insolation in sequential order) was finally selected (*p* < 0.05), with 18.0% explained variance (Fig. 3B). Next, two db-RDAs constrained to farming region and the best-fit agro-meteorological model were compared using Procrustes analysis, revealing two ordination plots that were very strongly correlated with one another (*r* = 0.9171, *p* = 0.001) (Fig. 3C). The db-RDA correlations were further tested after removing either core or unique ASVs. This analysis showed that db-RDAs excluding unique ASVs (*r* = 0.9223, *p* = 0.001) were more highly correlated than both db-RDAs excluding core ASVs (*r* = 0.7918, *p* = 0.001) and db-RDAs including all ASVs (*r* = 0.9171, *p* = 0.001) (Additional file 2: Fig. S3). These results prove that agro-meteorology has a large impact on the diversity of the fungal communities, particularly on core populations.

### Microbial networks are associated with the health of the host

**Fig. 4:**
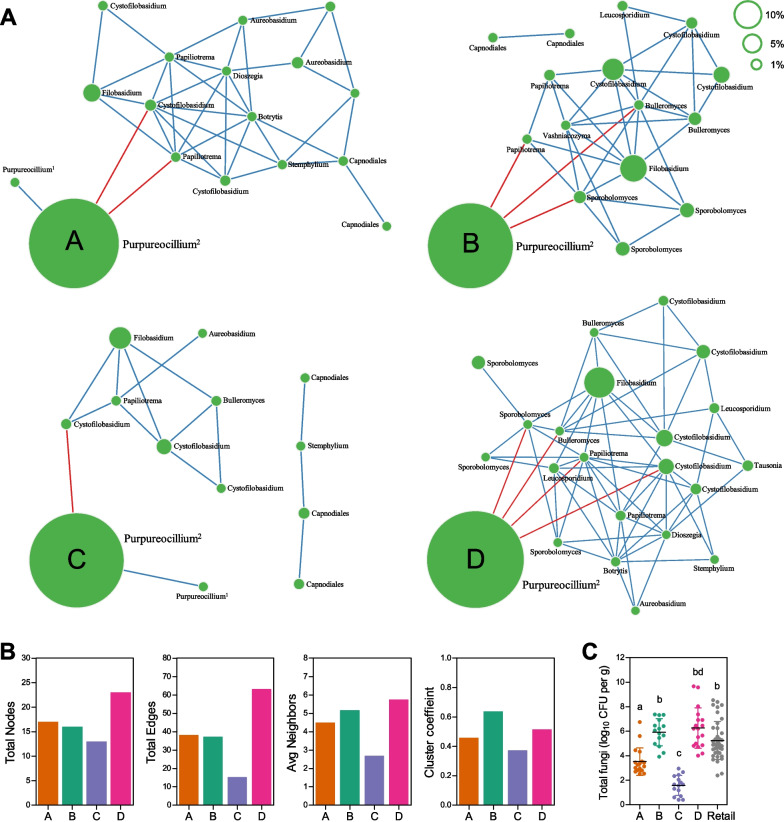
The microbial association networks of fungal communities of the farming regions. **A** Co-occurrence networks were constructed in terms of four regions. Red and blue lines indicate negative and positive correlations, respectively (*r* > 0.4, *P* < 0.01). The size of the node proportionally corresponds to relative abundance of ASVs. **B** Comparison of network properties between fungal communities of four regions. Yellow in the graph of total nodes indicates the number of nodes with > 1% average relative abundance. **C** Total viable fungi were compared by farming region. All data are mean ± s.d. Statistical significance was determined by one-way ANOVA with Tukey’s multiple comparison test

ASV co-occurrence was used to construct a microbial association network that inferred interspecies interactions of fungal populations [52]. In total, 95 correlations connecting 33 ASVs were predicted (*r* > 0.4 and *P* < 0.01), most of which were positive (91 positive and four negative correlations), showing that communities were dominated by fungal populations supporting each others’ fitness. Next, the microbial network was separated by farming region by selecting region-specific ASVs (> 0.03% average abundance and found in > 30% of regional samples). Four networks were established comprising 38, 37, 15, and 63 correlations connecting 17, 16, 13, and 22 ASVs for regions A, B, C, and D, respectively, and comprised two subnetworks (*Purpureocillium* and *Filobasidium*) that were negatively associated with one another (Fig. 4A). The region D network had the most microbial associations and the region C network had the least (Fig. 4B). The number of average neighbors per node was lowest in the region C network and was highest in the region B and D networks (Fig. 4B). Clustering coefficient for the average ratio of observed connections to all possible connections in nodes was higher in the region B and D networks than in the region A and C networks (Fig. 4B). These data indicate that microbial associations were simple and sparse in regions A and C, compared with regions B and D.

Weak associations between community members reflect a lack of community fitness in the host, leading to low total abundance of the whole community [53]. Because microbial biomass is historically used as an indicator of microbial perturbations [53–55], these relationships were verified by comparing total abundance of fungal communities using cultivation. Total number of fungi was the largest in regions B (5.93 ± 1.11 log CFUs per gram) and D (6.26 ± 1.65 log CFUs per gram), followed by region A (3.53 ± 1.12 log CFUs per gram), and region C (1.58 ± 0.82 log CFUs per gram) (*p* < 0.05) (Fig. 4C), showing that regional variation in the composition of fungal communities was expanded to differences in microbial associations and total quantity of the fungal communities. At the time of sampling, black rot and downy mildew occurred in region C before sampling, consistent with low floret weights in region C samples (approximately 250 g) compared with those of other regions (400 g). This was also consistent with total fungal abundance in healthy, retail broccoli (5.24 ± 1.55 log CFUs per gram) (*p* < 0.05) (Fig. 4C). Disease-associated damage likely reduced the carrying capacity of the host plants in region C, leading to the development of low density fungal communities with low network stability. Together, these results suggest that microbial networks and total abundance of fungal communities are associated with the health of the host plant in broccoli.

### Host development enriches the species of core genera in fungal communities

**Fig. 5:**
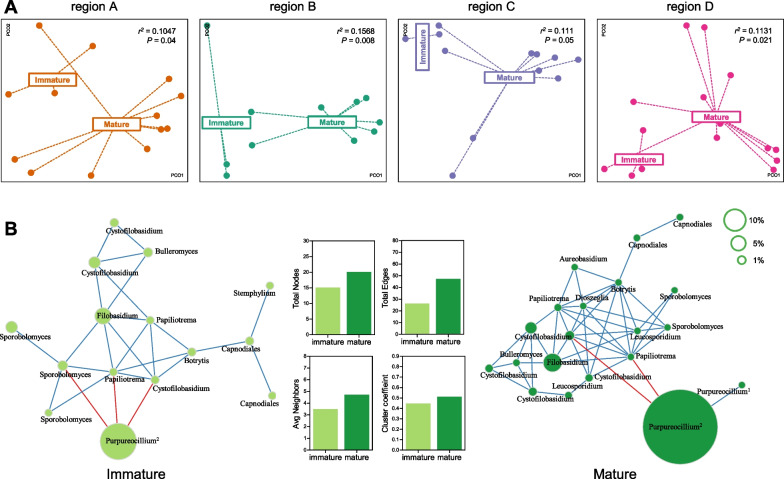
Fungal community composition in immature and mature broccoli samples. **A** The mature and immature communities of each region were compared separately using Principal coordinates Analysis based on Jaccard distance. Statistical significance was determined by PERMANOVA. **B** Co-occurrence networks were constructed in terms of maturity. Red and blue lines indicate negative and positive correlations, respectively (*r* > 0.4, *P* < 0.01). The size of the node proportionally corresponds to relative abundance of ASVs. Four network properties were compared between fungal networks of immature and mature communities

Broccoli plants from the initial sampling locations were cultivated for 6–7 fewer weeks than plants from other farms, and these samples were considered immature. Immature florets had lower mass (80–120 g) than florets from mature plants (250–400 g). Fungal communities were separated by farming region and compared according to host development. The fungal communities of immature samples significantly differed from those of mature samples across the regions in the PCoA plots with Bray–Curtis (Fig. 5A) and Jaccard distances (Additional file 1: Fig. S4A) (*p* < 0.05), except for region D with Bray–Curtis dissimilarity. These results suggest that the fungal community changes along with host development in field-grown broccoli.

To further investigate the interspecies associations within fungal communities, co-occurrence was examined in fungal ASVs and used to construct microbial networks. Microbial networks were separated according to sample maturity by using only group-specific ASVs (≥ 0.05% average abundance, > 30% samples). As illustrated in Fig. 4, similar network structures were exhibited in both samples (Fig. 5B). The immature community network comprised fewer nodes and edges than the mature community network (Fig. 5B). The number of average neighbors per node and clustering coefficient were also lower in the immature community network (Fig. 5B). These data further suggest that interactivity among community members increases with host development.

The effect size of developmental stage was significantly larger with Bray–Curtis dissimilarity (*r* = 0.2753 ± 0.1119) than with Jaccard distance (*r* = 0.1214 ± 0.0239) (two-tailed Student *t* test, *p* = 0.0360), and we therefore sought fungal ASVs that were differentially abundant between immature and mature samples using LEfSe analysis [43]. In total, 21 differentially abundant ASVs were identified (LDA score > 3.0, *p* < 0.05), four of which were enriched in mature samples, and 17 of which were enriched in immature samples (Additional file 1: Fig. S4B). The ASVs enriched in mature samples not only belonged to the core genera, but had an average abundance of ≥ 1%. ASVs enriched in immature samples had an average abundance of < 0.5% (14 ASVs) or were not from core genera (11 ASVs). In conclusion, fungal populations of core genera may be selectively enriched during host development.

### Purpureocillium is a key player in the assembly of broccoli mycobiota

The first axis of the unsupervised Bray–Curtis and Jaccard PCoA plots accounts for the largest variance of the broccoli mycobiota, but no correlations were found with farming conditions or host development. Specifically, the eigenvalues of the first axis of Bray–Curtis (3.8% of total variance) and Jaccard (4.5%) db-RDA constrained to farming region did not reach those of Bray–Curtis (41.6%) and Jaccard PCoA (7.7%). Similar results were seen for host development, where the first axis eigenvalues of db-RDA (8.6% for Bray–Curtis and 2.7% for Jaccard) constrained to developmental stage were even lower than those of the PCoA plots (41.9% for Bray–Curtis and 8.1% for Jaccard). To understand the source of the largest variation in the fungal communities, cluster numbers were optimized, where between-cluster distances were larger than within-cluster distances, using *k*-mean algorithm-based PAM clustering [40].

Two clusters were observed: clusters 1 and 2, with 20 (39.2%) and 31 samples (60.8%), respectively (Additional file 1: Fig. S5A). Using relative ASV abundance, the representative taxa of the two clusters were identified as *Filobasidium* ASV for cluster 1 and *Purpureocillium* ASV^2^ for cluster 2 (Additional file 1: Fig. S5B). The abundance of *Purpureocillium* ASV^2^ strongly correlated with sample variation along the first axis of the Bray–Curtis PCoA plot (*r* = 0.9801, *p* < 0.0001), and the abundance of the *Filobasidium* ASV was moderately correlated with the first axis of the Bray–Curtis PCoA plot (*r* = − 0.5934, *p* < 0.0001) (Additional file 1: Fig. S5C), confirming their negative relationship (*r* = − 0.5196, *p* < 0.0001), as indicated in the microbial networks (Fig. 4). No differences were observed in the total abundance of fungal communities (Additional file 1: Fig. S5D). However, Shannon diversity was higher in cluster 1 than in cluster 2, which seems attributable to an increase of Pielou’s evenness rather than to the number of ASVs (Additional file 1: Fig. S5E). This was consistent with the observed microbial networks, where the *Filobasidium* ASV was found to be co-abundant with all ASVs but the *Purpureocillium* ASVs was not.

**Fig. 6:**
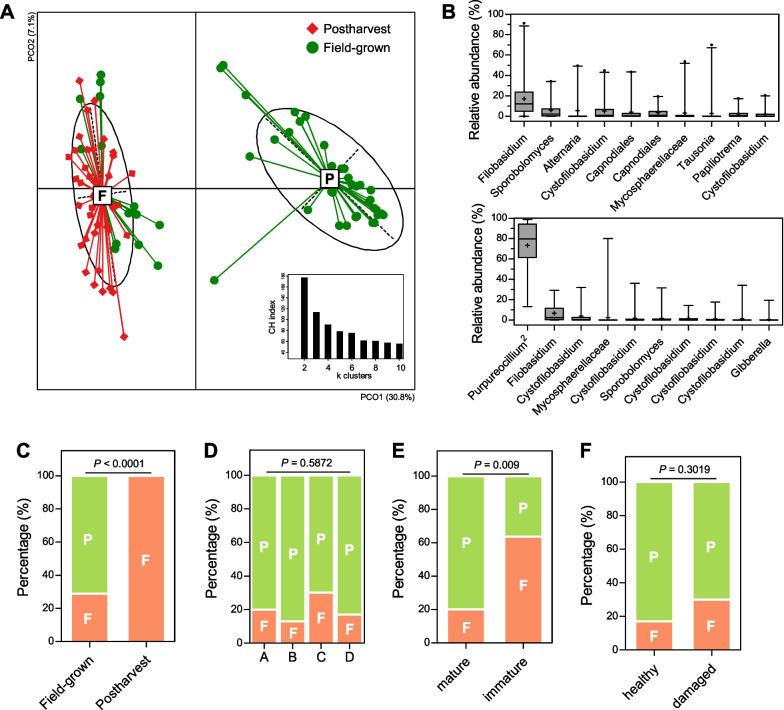
The global clustering of fungal communities of postharvest and field-grown broccoli. **A** The optimal number of clusters were defined based on Calinski-Harabasz index (lower right panel), and fungal communities were globally clustered into two groups (F for *Filobasidium* and P for *Purpureocillium*). Red diamond and green circle indicate postharvest and field-grown samples, respectively. **B** The abundant ASVs of *Filobasidium* (upper panel) and *Purpureocillium* (lower panel) types are shown using a rank abundance plot. Box and whisker plots are shown to 2.5% and 97.5%. The sample distribution of *Filobasidium* and *Purpureocillium* types was compared in terms of postharvest (**C**), farming region (**D**), maturity (**E**), and health (**F**). Statistical significance was evaluated using chi-square test, chi-square test for trend, or Fisher’s exact test

To investigate this further, a dataset from 37 postharvest samples was incorporated and the optimal number of clusters was reassessed [33]. Two clusters remained clearly identified (Fig. 6A), with the *Filobasidium* and *Purpureocillium*^2^ ASV still representative of each cluster (Fig. 6B). The two ASVs also remained negatively correlated with one another in the extended analysis (Spearman correlation, *r* = − 0.5175, *p* < 0.0001), confirming that an antagonistic relationship between *Purpureocillium* and *Filobasidium* caused the largest variation within the fungal communities. No difference was observed in total abundance of fungal communities (Additional file 1: Fig. S6A), and Shannon diversity supported by Pielou’s evenness was higher in the *Filobasidium* cluster than in the *Purpureocillium* cluster (Additional file 1: Fig. S6B). Thus, global comparison of the fungal communities demonstrated that the broccoli phyllosphere harbored two distinct types of fungal communities, dominated by either *Filobasidium* or *Purpureocillium*.

Fifty-two (59.1%) and 36 (40.9%) samples were attributed to the *Filobasidium* and *Purpureocillium* types, respectively. All the postharvest samples belonged to the *Filobasidium* type, and the *Purpureocillium* type was found only in field-grown samples (*p* < 0.0001) (Fig. 6C), suggesting that a shift from the *Purpureocillium* to *Filobasidium* type occurred after crop harvest. Second, the distribution of samples of the two types was independent of farming region, although the *Filobasidium* type was found more in regions A and C than in regions B and D (Fig. 6D). Third, 15 field-grown samples (29.4%) were included in the *Filobasidium* type, with immature samples more likely to be of this type than mature samples (*p* = 0.009) (Fig. 6E), indicating an association of the *Filobasidium* type with under-developed communities. Forth, the distribution of samples of the two types also seemed independent of host health (Fig. 6F), but the damaged samples most likely belong to the *Filobasidium* type after transforming into absolute abundance profiles (Fig. 4C). These observations suggest that two different types of broccoli mycobiota are associated with host-associated factors.

**Fig. 7:**
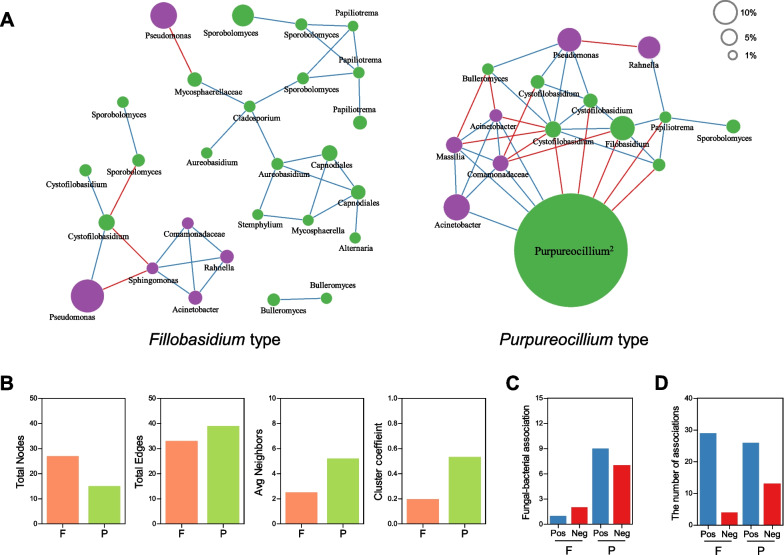
The microbial association networks of *Filobasidium* and *Purpureocillium* types. **A** Co-occurrence networks were constructed according to clustering. Red and blue lines indicate negative and positive correlations, respectively (*r* > 0.4, *P* < 0.01). Green and purple nodes indicate fungi and bacteria, respectively. The size of the node proportionally corresponds to relative abundance of ASVs. **B** Comparison of network properties between *Filobasidium* (F) and *Purpureocillium* (P) types. **C** Comparison of interspecies associations between *Filobasidium* (F) and *Purpureocillium* (P) types. **D** Comparison of the number of positive and negative associations between *Filobasidium* (F) and *Purpureocillium* (P) types

Last, microbial networks were constructed for the *Filobasidium* and *Purpureocillium* types using fungal and bacterial ASVs (> 30% samples per type, only field-grown samples). The 16S rRNA amplicon data of field-grown broccoli were re-analyzed [8], and bacterial ASVs were combined with fungal ASVs. Overall, the *Filobasidium* network of 15 field-grown samples was loose and sparse, whereas the *Purpureocillium* network was dense and interactive (Fig. 7A), as corroborated by the ratio of total edges to total nodes, average neighbor number, and clustering coefficient (Fig. 7B). Furthermore, the number of inter-kingdom associations (Fig. 7C) and the ratio of negative to positive associations (Fig. 7D) were higher in the *Purpureocillium* network than in the *Filobasidium* network. Overall, comparison of microbial networks demonstrated that the two types of broccoli mycobiota exhibited differences in robustness in their microbial networks [56].

## Discussion

Our study demonstrated that environment and host-associated sources of variation interact in complex ways to influence fungal communities in the phyllosphere, revealing the specificity, stability, and variability of phyllosphere mycobiota of field-grown broccoli. Fungal communities changed during host development from immature to mature plants, and also developed differently among diverse farming regions that varied in their agro-meteorological conditions. Upon disease-induced damages, the fungal communities experienced abnormal development in their overall community size and microbial network structure. Microbial co-occurrence networks illustrated the core phyllosphere mycobiota of field-grown broccoli, and mainly comprised members of six genera (*Purpureocillium*, *Filobasidium*, *Cystofilobasidium*, *Papiliotrema*, *Aureobasidium*, and unclassified genera of Capnodiales) that were not observed in other fresh fruits and vegetables [4, 10, 20, 47, 50, 57–59]. Using *Purpureocillium* spp. as an indicator, the broccoli mycobiota were classified into two distinct types, one of which was more frequently observed in immature, damaged, or postharvest samples.

The distinct fungal communities observed in different farming regions represented distinct responses to variation associated with diverse environmental conditions. The majority of fungal ASVs were unique to their farming region (54.8 ± 6.9% per region), and these were the major contributors to the regional variation of the fungal communities. In general, microbial dispersal from the surrounding environments such as soil [60] and near-surface atmosphere [61] were the main sources for the phyllosphere microbiota in plants. Of these, the phyllosphere microbiota is particularly subject to aerial sources through stochastic dispersal [61, 62], precipitation [63], and wind dust [9] during cultivation. Previous research found that leaves from plants grown in a glasshouse had low species richness compared with those from field-grown plants [64], providing additional evidence for the importance of aerial sources in the phyllosphere microbiota. While members of the phyllosphere microbiota are acquired initially from their surroundings, relatively few species are maintained and selectively enriched during plant maturation [61, 65], indicating the operation of niche-based deterministic processes during the assembly of phyllosphere microbiota. Region-specific unique ASVs were rare (2.2 ± 0.9%) and in very low abundance (0.02 ± 0.1%), suggesting that they may be transient and environmentally derived. Given that allochthonous microbes are likely to be dependent on the local environment of the sampling region, including climate [66], the association of fungal communities with regional agro-meteorology supports the hypothesis that unique microbiota are locally derived. Thus, a large proportion of region-specific, unique mycobiota may reflect the importance of the specific field environment in the formation of phyllosphere mycobiota on field-grown broccoli.

The prevalent and abundant fungal ASVs also contributed to regional variation of the phyllosphere mycobiota. Variations in the abundance of three populations of the core genera (*Cystofilobasidium* and *Purpureocillium*) were significantly correlated with regional differences, and the composition of the core ASVs strongly co-varied with agro-meteorological factors, suggesting that the core mycobiota of field-grown broccoli may be assembled preferentially by a local niche-based deterministic process. Interestingly, soil moisture and soil temperature were identified as contributing attributes to explain regional variation in the fungal communities. For example, the relatively high percentage of soil moisture in region D separated its fungal communities from those from other regions (20.4% of total variance, *P* = 0.002). This finding is consistent with Zarraonaindia et al. [67], who described the two main factors driving leaf microbiota diversity in grapevine. First, nearly 80% of the soils in Jeju Island are covered by volcanic ash soils, Andisols, which contain a large quantity of Si that readily dissolves in soil solutions [68]. The central and southeastern regions (regions B and D) of the island are covered by Andisols, whereas the coastal and middle areas of the western and northern regions (region A, B and C) mainly contain non-Andisols [69]. Second, annual precipitation is high in the central and southeastern regions of the island, whereas precipitation is relatively low and evapotranspiration is high in the coastal and middle-mountainous areas of the western and northern regions [68]. Together, these data indicate that microbial community assembly in the broccoli phyllosphere was influenced by regional variation in edaphic and meteorological properties throughout the sampling area [64, 70]. Collectively, habitat-dependent variation is present in the fungal communities of field-grown broccoli as well as in the bacterial communities [8].

*Purpureocillium* spp. are core components of fungal communities on field-grown broccoli across a range of geographical, developmental, and physical conditions. *Purpureocillium* ASVs were observed predominantly (82.5%) and abundantly (59.7 ± 35.7%) in mature samples, and *Purpureocillium* was the most influential taxon discriminating the fungal communities. *Purpureocillium* spp. are endophytic plant colonizers [71] that have plant growth promoting effects [72], yet the dominance of *Purpureocillium* spp. in plant-associated fungal communities has not been previously reported in plants, including fresh vegetables. Notably, lower *Purpureocillium* colonization was associated with higher levels of network instability, as in immature and postharvest samples, suggesting a potential role for *Purpureocillium* spp. in establishing healthy mycobiota in the broccoli phyllosphere. *Purpureocillium* was detected in less than a half (45.4%) of immature samples, and the abundance of *Purpureocillium* increased during development from immature (23.8 ± 38.2%) to mature (59.5 ± 35.6%) plants, suggesting mutual interactions between *Purpureocillium* and the host during cultivation. *Purpureocillium* spp. are frequently detected in the rhizosphere of wide range of crops [72], suggesting that the rhizosphere might influence the colonization of *Purpureocillium* during the early stages of broccoli development. However, the ecology of *Purpureocillium* spp. remains unclear and requires further elucidation. For example, different broccoli samples originating from the same farm sometimes had very different *Purpureocillium* spp. levels (Additional file 1: Fig. S6C), indicating high inter-sample variability in the abundance of *Purpureocillium* spp. on broccoli grown under the same cultivation conditions. This inter-sample variation may be driven by hitherto unrecognized smaller-scale factors impacting the crop in addition to larger-scale factors such as host identity, farming location, and agricultural method.

All genera of the *Fillobasidium* subnetwork had negative relationships with *Purpureocillium*. Microbial network analysis found that five core genera (*Filobasidium*, *Cystofilobasidium*, *Papiliotrema*, *Aureobasidium*, and *Capnodiales*) were the main components of the *Fillobasidium* subnetwork across diverse geographical, developmental, and physical conditions. By contrast, genera that were neighbors to the core genera were diverse across different conditions. For example, *Bulleromyces* was found in regions A, B, and D, and *Sporobolomyces* and *Leucosporidium* were found in regions B and D. *Aureobasidium* and *Leucosporidium* were enriched in mature communities compared with immature communities. Additional fungal genera such as *Stemphylium*, *Vishniacozyma*, *Dioszegia*, and *Tausonia* were sometimes observed as neighbors. At the species-level, all members had positive relationships with one another, indicating sharing of niches and resources and/or positive interspecies interactions. However, the *Filobasidium* subnetwork became looser and less complex in the absence of *Purpureocillium*, predicting that their positive relationship may represent high niche and/or resource overlap, rather than cooperative interactions. Hernandez et al. [56] demonstrated that the dominance of positive associations can easily propagate perturbations in a community through positive feedback loops, resulting in unstable communities with lower network connectivity. In this study, the *Filobasidium* subnetwork exhibited substantial variability under different host and agricultural conditions. During maturation, the microbial networks of fungal communities became more complex; however, complexity was reduced after disease occurrence. The connectivity of *Filobasidium* subnetwork members contributed to both these situations. Communities dominated by positive co-occurrences likely accounts for the high variability of the *Filobasidium* subnetwork compared with the *Purpureocillium* subnetwork. Low complexity of interspecies associations also coincided with low abundance of fungal communities. Given that microbial community size is used as a proxy for overall microbial activities [53, 54], this suggests that a decrease in host carrying capacity may not support the growth of *Filobasidium* subnetwork members after disease occurrence. This was also consistent with the Hernandez study [56], which firstly demonstrated that network complexity decreased with microbial abundance and diversity in unstable microbial communities, suggesting a relationship between microbiota stability and network properties in the phyllosphere ecosystem. Collectively, interspecies associations of the *Filobasidium* subnetwork could be indicative of microbial perturbations and of host quality in field-grown broccoli, underscoring the importance of microbe–microbe and host–microbiota interactions in understanding the ecology of the phyllosphere mycobiota.

Microbiota on the surfaces of fresh vegetables potentially increase vegetable degradation and decomposition [15]. Indigenous pathogens present in harvested vegetables, rather than external microorganisms, have been recently examined as spoilage organisms [3, 20, 49, 73–75]. Correspondingly, the observation of plant pathogenic *Alternaria* and *Botrytis* spp. in the original broccoli mycobiota supports the importance of investigating the preharvest microbiota of fresh vegetables. The original community is vulnerable to postharvest changes such as deficiency in host controls and long-term storage at low temperature, and alterations in the original community are likely to facilitate the development of indigenous pathogens [70]. For instance, we recently reported enrichment of *Alternaria* spp. from preharvest to postharvest [33]. Further studies are needed to determine whether the enrichment of such genera necessarily result in disease development, or whether the observed species or strains of such genera are indeed pathogenic to fresh vegetables. Furthermore, construction of agonistic and antagonistic associations in preharvest fungal populations may provide an opportunity to identify putative biocontrol agents for indigenous pathogens.

## Conclusion

The phyllosphere microbiota is a newly-emerging factor capable of influencing quality of agricultural crops, including fresh vegetables, and the diversity and function of phyllosphere microbiota should be considered within the context of the agricultural landscape. A large variety of environmental, agricultural, and host conditions influence the formation of phyllosphere microbiota [24], and preharvest microbiota persist through harvest, during postharvest storage, and finally to the consumer [11]. This study determined the main structure of preharvest broccoli mycobiota across a range of environmental, agricultural, and host conditions, and highlighted the importance and functionality of constructing microbial associations to complement microbial composition data and disentangle the diversity of fungal communities in the phyllosphere of fresh vegetables [76]. Further in-depth analysis encompassing a wide variety of fresh vegetables would enhance our knowledge of the phyllosphere mycobiota of fresh vegetables and provide insight into the ecological drivers of the phyllosphere mycobiota. Understanding phyllosphere mycobiota networks will also facilitate the development of biocontrol strategies aimed at strengthening plant health and stabilizing crop production upon exposure to biotic and abiotic stresses.

## Supplementary Information

**Additional file 1: Fig. S1.** The images of the farms, broccoli plants and flower heads are provided (Kim et al., 2018). **Fig. S2**. (A) The statistics of pairwise comparison among the fungal communities of the farming regions on the basis of Bray-Curtis dissimilarity and Jaccard distance. Statistical significance was evaluated with Bray-Curtis dissimilarity using PERMANOVA (B) Comparison of relative abundance of core and unique ASVs in the farming regions. Statistical significance was evaluated using two-tailed Mann-Whitney U test. (C) The relative abundance of three discriminant ASVs for regional variation were shown. **Fig. S3**. Community-level associations between Jaccard distance-based db-RDA plots constrained to farming region and six selected agrometeorological factors were performed using Procrustes analysis. The db-RDA plots constrained to farming region were generated by excluding either core ASVs (A) or unique ASVs (B). **Fig. S4**. (A) Immature and mature fungal communities were compared by using Principal coordinates Analysis based on Jaccard distance. (B) Effect size scores of linear discriminant analysis calculated for differences in fungal ASVs abundance between mature and immature samples (logarithmic LDA score >3.0). **Fig. S5**. The clustering of fungal communities of field-grown broccoli. (A) The optimal number of clusters were defined based on Calinski-Harabasz index (lower right panel), and fungal communities were clustered into two groups. Two ASVs that had the strongest correlations with the first axis of PCoA were shown (r > 0.5, P < 0.01). (B) The abundant ASVs of cluster 1 (upper panel) and 2 (lower panel) types are shown using a rank abundance plot. Box and whisker plots are shown to min and max. (C) The relative abundances of Purpureocillium and Filobasidium ASVs were correlated with the first axis of unsupervised PCoA plot for Bray-Curtis dissimilarity. Statistical significance was determined by Pearson correlation. Total number of viable fungi (D) and diversity indices (E) of fungal communities of the two clusters were compared. All data are mean ± SD. Statistical significance was determined by two-tailed Mann-Whitney U test. **Fig. S6**. Total number of viable fungi and bacteria (A), and fungal diversity indices (B) were compared between Filobasidium and Purpureocillium types. All data are mean ± SD. Statistical significance was determined by two-tailed Mann-Whitney U test. (C) The abundance of the Purpureocillium ASV in the communities of four farming regions. Dots colored in red, orange, green, and purple indicate samples originated from the same farm.

**Additional file 2: Table S1.** The information of sampling locations. **Table S2.** Sequence processing.

## Data Availability

The EMBL-EBI accession number for the ITS2 gene sequences is PRJEB39485.

## References

[1] Vorholt JA (2012). Microbial life in the phyllosphere. Nat Rev Microbiol.

[2] Cordovez V, Dini-Andreote F, Carrion VJ, Raaijmakers JM (2019). Ecology and evolution of plant microbiomes. Ann Rev Microbiol.

[3] Shi Y, Yang Q, Zhao Q, Dhanasekaran S, Ahima J, Zhang X, Zhou S, Droby S, Zhang H (2022). *Aureobasidium pullulans* S-2 reduced the disease incidence of tomato by influencing the postharvest microbiome during storage. Postharvest Biol Technol.

[4] Zhimo VY, Kumar A, Biasi A, Salim S, Feygenberg O, Toamy MA, Abdelfattaah A, Medina S, Freilich S, Wisniewski M (2021). Compositional shifts in the strawberry fruit microbiome in response to near-harvest application of *Metschnikowia fructicola*, a yeast biocontrol agent. Postharvest Biol Technol.

[5] Shen Y, Nie J, Kuang L, Zhang J, Li H (2020). DNA sequencing, genomes and genetic markers of microbes on fruits and vegetables. Microbial Biotechnol.

[6] Aw TG, Wengert S, Rose JB (2016). Metagenomic analysis of viruses associated with field-grown and retail lettuce identifies human and animal viruses. Int J Food Microbiol.

[7] Dees MW, Lysoe E, Nordskog B, Brurberg MB (2015). Bacterial communities associated with surfaces of leafy greens: shift in composition and decrease in richness over time. Appl Environ Microbiol.

[8] Kim MS, Bae JW, Park EJ (2018). Geographic and host-associated variations in bacterial communities on the floret surfaces of field-grown broccoli. Appl Environ Microbiol.

[9] Rastogi G, Sbodio A, Tech JJ, Suslow TV, Coaker GL, Leveau JHJ (2012). Leaf microbiota in an agroecosystem: spatiotemporal variation in bacterial community composition on field-grown lettuce. ISME J.

[10] Karlsson I, Friberg H, Kolseth AK, Steinberg C, Persson P (2017). Organic farming increases richness of fungal taxa in the wheat phyllosphere. Mol Ecol.

[11] Leff JW, Fierer N (2013). Bacterial communities associated with the surfaces of fresh fruits and vegetables. PLoS ONE.

[12] Williams TR, Moyne AL, Harris LJ, Marco ML (2013). Season, irrigation, leaf age, and *Escherichia coli* inoculation influence the bacterial diversity in the lettuce phyllosphere. PLoS ONE.

[13] Rastogi G, Coaker GL, Leveau JHJ (2013). New insights into the structure and function of phyllosphere microbiota through high-throughput molecular approaches. FEMS Microbiol Lett.

[14] FAO. Food Losses and Waste 2015.

[15] Kusstatscher P, Cernava T, Abdelfattah A, Gokul J, Korsten L, Berg G (2020). Microbiome approaches provide the key to biologically control postharvest pathogens and storability of fruits and vegetables. FEMS Microbiol Ecol.

[16] Pitt JI, Hocking AD. The ecology of fungal food spoilage. In: Fungi and Food Spoilage. Springer; 2009. p. 3–9.

[17] Snyder AB, Churey JJ, Worobo RW (2019). Association of fungal genera from spoiled processed foods with physicochemical food properties and processing conditions. Food Microbiol.

[18] Manthou E, Coeuret G, Chaillou S, Nychas GE (2021). Evolution of fungal community associated with ready-to-eat pineapple during storage under different temperature conditions. Food Microbiol.

[19] Buchholz F, Kostic T, Sessitsch A, Mitter B (2018). The potential of plant microbiota in reducing postharvest food loss. Microbial Biotechnol.

[20] Chen J, Yan R, Hu Y, Zhang N, Hu H (2019). Compositional shifts in the fungal diversity of garlic scapes during postharvest transportation and cold storage. LWT-Food Sci Technol.

[21] Berg G, Köberl M, Rybakova D, Müller H, Grosch R, Smalla K. Plant microbial diversity is suggested as the key to future biocontrol and health trends. FEMS Microbiol Ecol. 2017;93. 10.1093/femsec/fix050 28430944

[22] Leneveu-Jenvrin C, Charles F, Barba FJ, Remize F (2020). Role of biological control agents and physical treatments in maintaining the quality of fresh and minimally-processed fruit and vegetables. Crit Rev Food Sci Nutr.

[23] Wisniewski M, Droby S (2019). The postharvest microbiome: the other half of sustainability. Biol Control.

[24] Dastogeer KMG, Tumpa FH, Sultana A, Akter MA, Chakraborty A (2020). Plant microbiome–an account of the factors that shape community composition and diversity. Curr Plant Biol.

[25] Agler MT, Ruhe J, Kroll S, Morhenn C, Kim ST, Weigel D, Kemen EM (2016). Microbial hub taxa link host and abiotic factors to plant microbiome variation. PLoS Biol.

[26] Neu AT, Allen EE, Roy K (2021). Defining and quantifying the core microbiome: challenges and prospects. Proc Natl Acad Sci USA.

[27] Rottjers L, Faust K (2018). From hairballs to hypotheses-biological insights from microbial networks. FEMS Microbiol Rev.

[28] Kinkel LL, Bakker MG, Schlatter DC (2011). A coevolutionary framework for managing disease-suppressive soils. Annu Rev Phytopathol.

[29] MAFRA: Statistics on production of greenhouse vegetable and greenhouse facilities for vegetable from Ministry of Agriculture, Food and Rural Affairs. 2012.

[30] GARES: Broccoli cultivation (http://www.agri.jeju.kr/jeju/technologycenter/technology/vegetable.htm?page=8&act=view&seq=26075) from the Agricultural Research and Extension Services in Jeju. 2012.

[31] Kim MS, Bae JW, Park EJ (2018). Postharvest processing decreases the richness of bacterial taxa in the phyllosphere of broccoli. J Appl Microbiol.

[32] Op De Beeck M, Lievens B, Busschaert P, Declerck S, Vangronsveld J, Colpaert JV (2014). Comparison and validation of some ITS primer pairs useful for fungal metabarcoding studies. PLoS ONE.

[33] Kim MS, Park EJ (2021). Postharvest-induced microbiota remodeling increases fungal diversity in the phyllosphere mycobiota of broccoli florets. Postharvest Biol Technol.

[34] Bolyen E, Rideout JR, Dillon MR, Bokulich NA, Abnet CC, Al-Ghalith GA, Alexander H, Alm EJ, Arumugam M, Asnicar F (2019). Reproducible, interactive, scalable and extensible microbiome data science using QIIME 2. Nat Biotechnol.

[35] Rivers AR, Weber KC, Gardner TG, Liu S, Armstrong SD (2018). ITSxpress: Software to rapidly trim internally transcribed spacer sequences with quality scores for marker gene analysis. F1000Res.

[36] Nilsson RH, Ryberg M, Abarenkov K, Sjokvist E, Kristiansson E (2009). The ITS region as a target for characterization of fungal communities using emerging sequencing technologies. FEMS Microbiol Lett.

[37] Callahan BJ, McMurdie PJ, Rosen MJ, Han AW, Johnson AJ, Holmes SP (2016). DADA2: High-resolution sample inference from Illumina amplicon data. Nat Meth.

[38] Nilsson RH, Larsson KH, Taylor AFS, Bengtsson-Palme J, Jeppesen TS, Schigel D, Kennedy P, Picard K, Glockner FO, Tedersoo L (2019). The UNITE database for molecular identification of fungi: handling dark taxa and parallel taxonomic classifications. Nucleic Acids Res.

[39] Kim MS, Hwang SS, Park EJ, Bae JW (2013). Strict vegetarian diet improves the risk factors associated with metabolic diseases by modulating gut microbiota and reducing intestinal inflammation. Environ Microbiol Rep.

[40] Arumugam M, Raes J, Pelletier E, Le Paslier D, Yamada T, Mende DR, Fernandes GR, Tap J, Bruls T, Batto JM (2011). Enterotypes of the human gut microbiome. Nature.

[41] Schwager E, Bielski C, Weingart G. ccrepe: ccrepe_and_nc.score. R package version 1.24.0. 2020.

[42] Dixon P (2003). VEGAN, a package of R functions for community ecology. J Veg Sci.

[43] Segata N, Izard J, Waldron L, Gevers D, Miropolsky L, Garrett WS, Huttenhower C (2011). Metagenomic biomarker discovery and explanation. Genome Biol.

[44] Gomba A, Chidamba L, Korsten L (2017). Effect of postharvest practices including degreening on citrus carpoplane microbial biomes. J Appl Microbiol.

[45] Zhang Q, Shi W, Zhou B, Du H, Xi L, Zou M, Zou H, Xin L, Gao Z, Chen Y (2021). Variable characteristics of microbial communities on the surface of sweet cherries under different storage conditions. Postharvest Biol Technol.

[46] Al-Bulushi IM, Bani-Uraba MS, Guizani NS, Al-Khusaibi MK, Al-Sadi AM (2017). Illumina MiSeq sequencing analysis of fungal diversity in stored dates. BMC Microbiol.

[47] Kusstatscher P, Zachow C, Harms K, Maier J, Eigner H, Berg G, Cernava T (2019). Microbiome-driven identification of microbial indicators for postharvest diseases of sugar beets. Microbiome.

[48] Diskin S, Feygenberg O, Maurer D, Droby S, Prusky D, Alkan N (2017). Microbiome alterations are correlated with occurrence of postharvest stem-end rot in mango fruit. Phytobiomes J.

[49] Zambounis A, Ganopoulos I, Tsaftaris A, Valasiadis D, Madesis P (2020). Metagenomics analysis of fungal communities associated with postharvest diseases in pear fruits under the effect of management practices. Arch Microbiol.

[50] Darlison J, Mogren L, Rosberg AK, Gruden M, Minet A, Line C, Mieli M, Bengtsson T, Hakansson A, Uhlig E (2019). Leaf mineral content govern microbial community structure in the phyllosphere of spinach (*Spinacia oleracea*) and rocket (*Diplotaxis tenuifolia*). Sci Total Environ.

[51] Abdelfattah A, Wisniewski M, Droby S, Schena L (2016). Spatial and compositional variation in the fungal communities of organic and conventionally grown apple fruit at the consumer point-of-purchase. Horticulture Res.

[52] Faust K, Raes J (2012). Microbial interactions: from networks to models. Nat Rev Microbiol.

[53] Contijoch EJ, Britton GJ, Yang C, Mogno I, Li Z, Ng R, Llewellyn SR, Hira S, Johnson C, Rabinowitz KM (2019). Gut microbiota density influences host physiology and is shaped by host and microbial factors. Elife.

[54] Vandeputte D, Kathagen G, D'Hoe K, Vieira-Silva S, Valles-Colomer M, Sabino J, Wang J, Tito RY, De Commer L, Darzi Y (2017). Quantitative microbiome profiling links gut community variation to microbial load. Nature.

[55] Tkacz A, Hortala M, Poole PS (2018). Absolute quantitation of microbiota abundance in environmental samples. Microbiome.

[56] Hernandez DJ, David AS, Menges ES, Searcy CA, Afkhami ME (2021). Environmental stress destabilizes microbial networks. ISME J.

[57] Sapkota R, Knorr K, Jørgensen LN, O'Hanlon KA, Nicolaisen M (2015). Host genotype is an important determinant of the cereal phyllosphere mycobiome. New Phytol.

[58] Chen W, Turkington TK, Lévesque CA, Bamforth JM, Patrick SK, Lewis CT, Chapados JT, Gaba D, Tittlemier SA, MacLeod A (2016). Geography and agronomical practices drive diversification of the epiphytic mycoflora associated with barley and its malt end product in western Canada. Agric Ecosyst Environ.

[59] Angeli D, Sare AR, Jijakli MH, Pertot I, Massart S (2019). Insights gained from metagenomic shotgun sequencing of apple fruit epiphytic microbiota. Postharvest Biol Technol.

[60] Grady KL, Sorensen JW, Stopnisek N, Guittar J, Shade A (2019). Assembly and seasonality of core phyllosphere microbiota on perennial biofuel crops. Nat Commun.

[61] Maignien L, DeForce EA, Chafee ME, Eren AM, Simmons SL (2014). Ecological succession and stochastic variation in the assembly of *Arabidopsis thaliana* phyllosphere communities. MBio.

[62] Levetin E, Dorsey K (2006). Contribution of leaf surface fungi to the air spora. Aerobiologia Bolongna..

[63] Llontop MEM, Tian L, Sharma P, Heflin L, Bernal-Galeano V, Haak DC, Clarke CR, Vinatzer BA (2021). Experimental evidence pointing to rain as a reservoir of tomato phyllosphere microbiota. Phytobiomes J.

[64] Birt HWG, Pattison AB, Skarshewski A, Daniells J, Raghavendra A, Dennis PG (2022). The core bacterial microbiome of banana (*Musa* spp.). Environ Microbiome..

[65] Copeland JK, Yuan L, Layeghifard M, Wang PW, Guttman DS (2015). Seasonal community succession of the phyllosphere microbiome. Mol Plant Microbe Interact.

[66] Redondo MA, Oliva J, Elfstrand M, Boberg J, Capador-Barreto HD, Karlsson B, Berlin A (2022). Host genotype interacts with aerial spore communities and influences the needle mycobiome of Norway spruce. Environ Microbiol.

[67] Zarraonaindia I, Owens SM, Weisenhorn P, West K, Hampton-Marcell J, Lax S, Bokulich NA, Mills DA, Martin G, Taghavi S (2015). The soil microbiome influences grapevine-associated microbiota. MBio.

[68] Park WP, Song KC, Koo BJ, Hyun HN. Distribution of available silicon of volcanic ash soils in Jeju island. Appl Environ Soil Sci. 2019;2729694.

[69] Park WP, Hyun HN, Koo BJ (2020). Silicon fractionation of soluble silicon in volcanic ash soils that may affect groundwater silicon content on Jeju Island. Korea Water.

[70] Wagner MR, Lundberg DS, Del Rio TG, Tringe SG, Dangl JL, Mitchell-Olds T (2016). Host genotype and age shape the leaf and root microbiomes of a wild perennial plant. Nat Commun.

[71] Tian XL, Cao LX, Tan HM, Zeng QG, Jia YY, Han WQ, Zhou SN (2004). Study on the communities of endophytic fungi and endophytic actinomycetes from rice and their antipathogenic activities in vitro. World J Microbiol Biotechnol.

[72] Baron NC, de Souza PA, Rigobelo EC (2020). *Purpureocillium lilacinum* and *Metarhizium marquandii* as plant growth-promoting fungi. PeerJ.

[73] Shen Y, Nie J, Dong Y, Kuang L, Li Y, Zhang J (2018). Compositional shifts in the surface fungal communities of apple fruits during cold storage. Postharvest Biol Tec.

[74] Bosch Y, Britt E, Perren S, Naef A, Frey JE, Buhlmann A (2021). Dynamics of the apple fruit microbiome after harvest and implications for fruit quality. Microorganisms.

[75] Yurgel SN, Abbey L, Loomer N, Gillis-Madden R, Mammoliti M (2018). Microbial communities associated with storage onion. Phytobiomes J.

[76] Karimi B, Maron PA, Boure NC-P, Bernard N, Gilbert D, Ranjard L (2017). Microbial diversity and ecological networks as indicators of environmental quality. Environ Chem Lett.

