# Supplementary material for: Composition and variability of core phyllosphere fungal mycobiota on field-grown broccoli
Source: Environ Microbiome. 2023 Mar 1;18:15. doi: 10.1186/s40793-023-00474-0 (PMC9976476; doi:10.1186/s40793-023-00474-0)
Supplement: Supplementary file 1 — Additional file 1: Fig. S1. The images of the farms, broccoli plants and flower heads are provided (Kim et al., 2018). Fig. S2. (A) The statistics of pairwise comparison among the fungal communities of the farming regions on the basis of Bray-Curtis dissimilarity and Jaccard distance. Statistical significance was evaluated with Bray-Curtis dissimilarity using PERMANOVA (B) Comparison of relative abundance of core and unique ASVs in the farming regions. Statistical significance was evaluated using two-tailed Mann-Whitney U test. (C) The relative abundance of three discriminant ASVs for regional variation were shown. Fig. S3. Community-level associations between Jaccard distance-based db-RDA plots constrained to farming region and six selected agrometeorological factors were performed using Procrustes analysis. The db-RDA plots constrained to farming region were generated by excluding either core ASVs (A) or unique ASVs (B). Fig. S4. (A) Immature and mature fungal communities were compared by using Principal coordinates Analysis based on Jaccard distance. (B) Effect size scores of linear discriminant analysis calculated for differences in fungal ASVs abundance between mature and immature samples (logarithmic LDA score >3.0). Fig. S5. The clustering of fungal communities of field-grown broccoli. (A) The optimal number of clusters were defined based on Calinski-Harabasz index (lower right panel), and fungal communities were clustered into two groups. Two ASVs that had the strongest correlations with the first axis of PCoA were shown (r > 0.5, P < 0.01). (B) The abundant ASVs of cluster 1 (upper panel) and 2 (lower panel) types are shown using a rank abundance plot. Box and whisker plots are shown to min and max. (C) The relative abundances of Purpureocillium and Filobasidium ASVs were correlated with the first axis of unsupervised PCoA plot for Bray-Curtis dissimilarity. Statistical significance was determined by Pearson correlation. Total number of viable fungi (D) and [file 40793_2023_474_MOESM1_ESM.pdf]

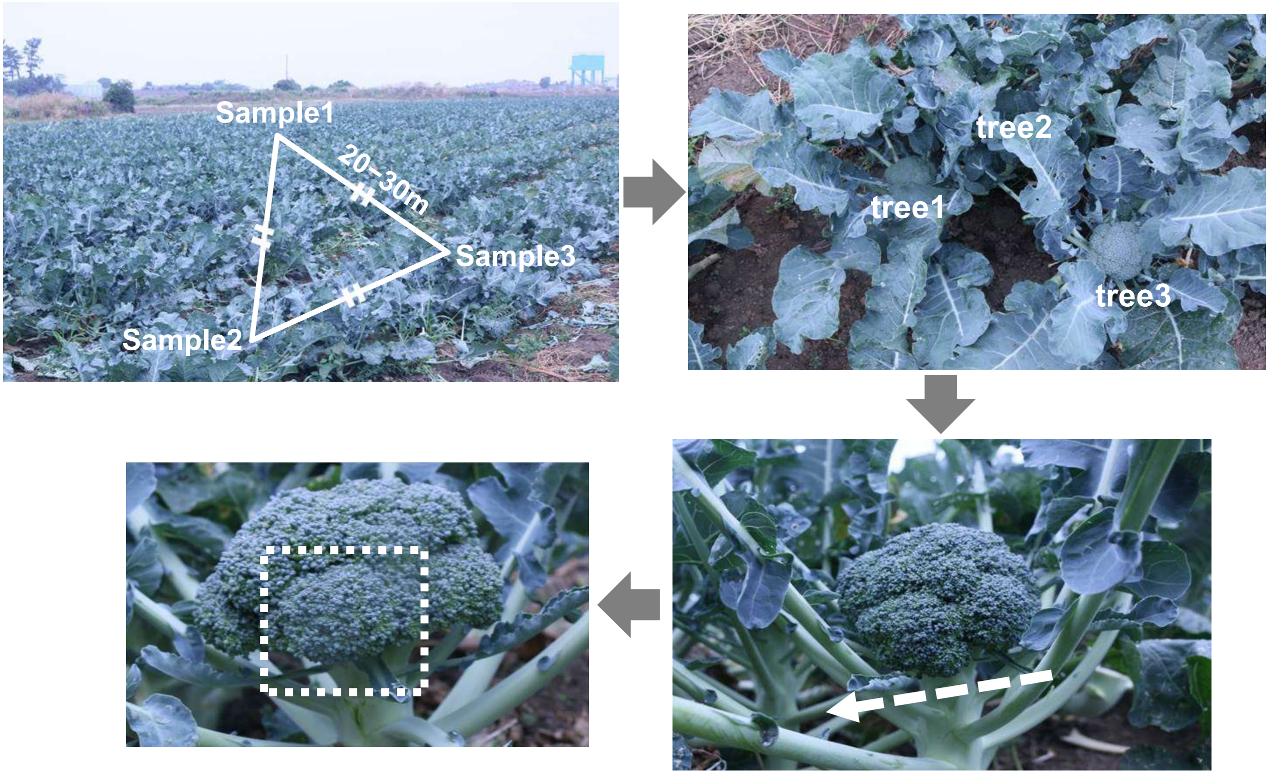

Fig. S1 The images of the farms, broccoli plants and flower heads are provided (Kim et al., 2018).

A

Variable	Bray-Curtis		Jaccard	
	R-square	*P*-value	R-square	*P*-value
Total	0.0715	0.209	0.1071	0.001
A vs. B	0.0729	0.085	0.0865	0.001
A vs. C	0.0304	0.617	0.0555	0.026
A vs. D	0.0576	0.133	0.0773	0.001
B vs. C	0.0397	0.456	0.0707	0.002
B vs. D	0.0636	0.140	0.0777	0.001
C vs. D	0.0430	0.312	0.0754	0.001

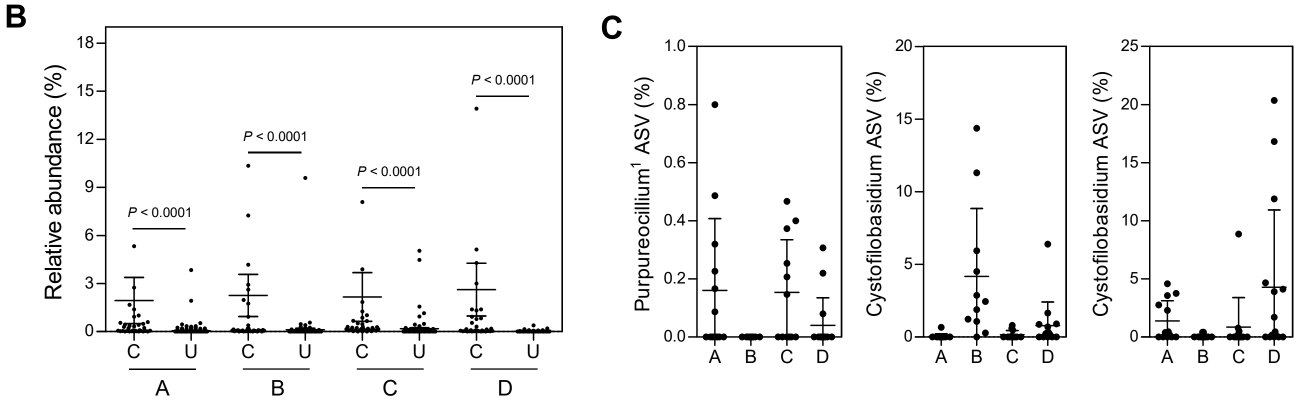

Fig. S2 (A) The statistics of pairwise comparison among the fungal communities of the farming regions on the basis of Bray-Curtis dissimilarity and Jaccard distance. Statistical significance was evaluated with Bray-Curtis dissimilarity using PERMANOVA (B) Comparison of relative abundance of core and unique ASVs in the farming regions. Statistical significance was evaluated using two-tailed Mann-Whitney U test. (C) The relative abundance of three discriminant ASVs for regional variation were shown.

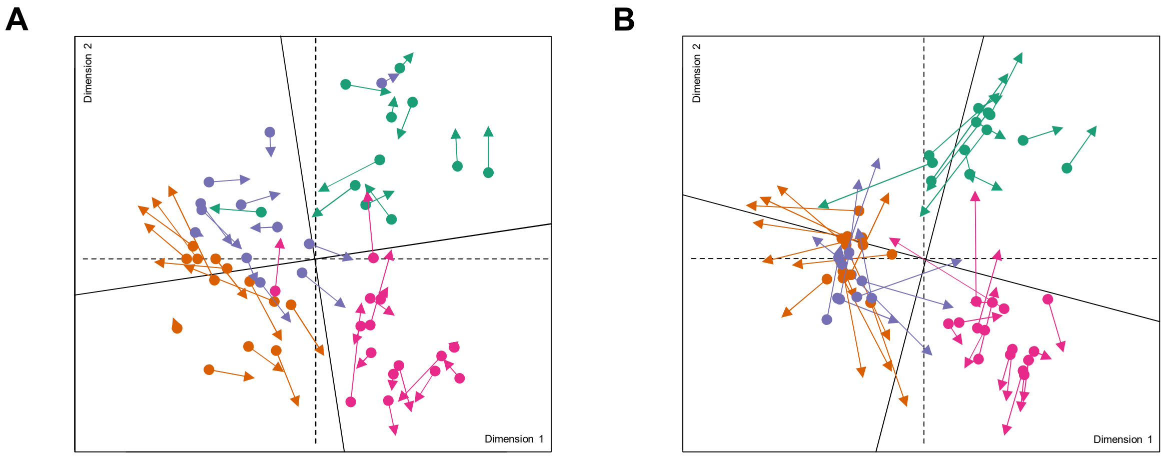

Fig. S3 Community-level associations between Jaccard distance-based db-RDA plots constrained to farming region and six selected agrometeorological factors were performed using Procrustes analysis. The db-RDA plots constrained to farming region were generated by excluding either core ASVs (A) or unique ASVs (B).

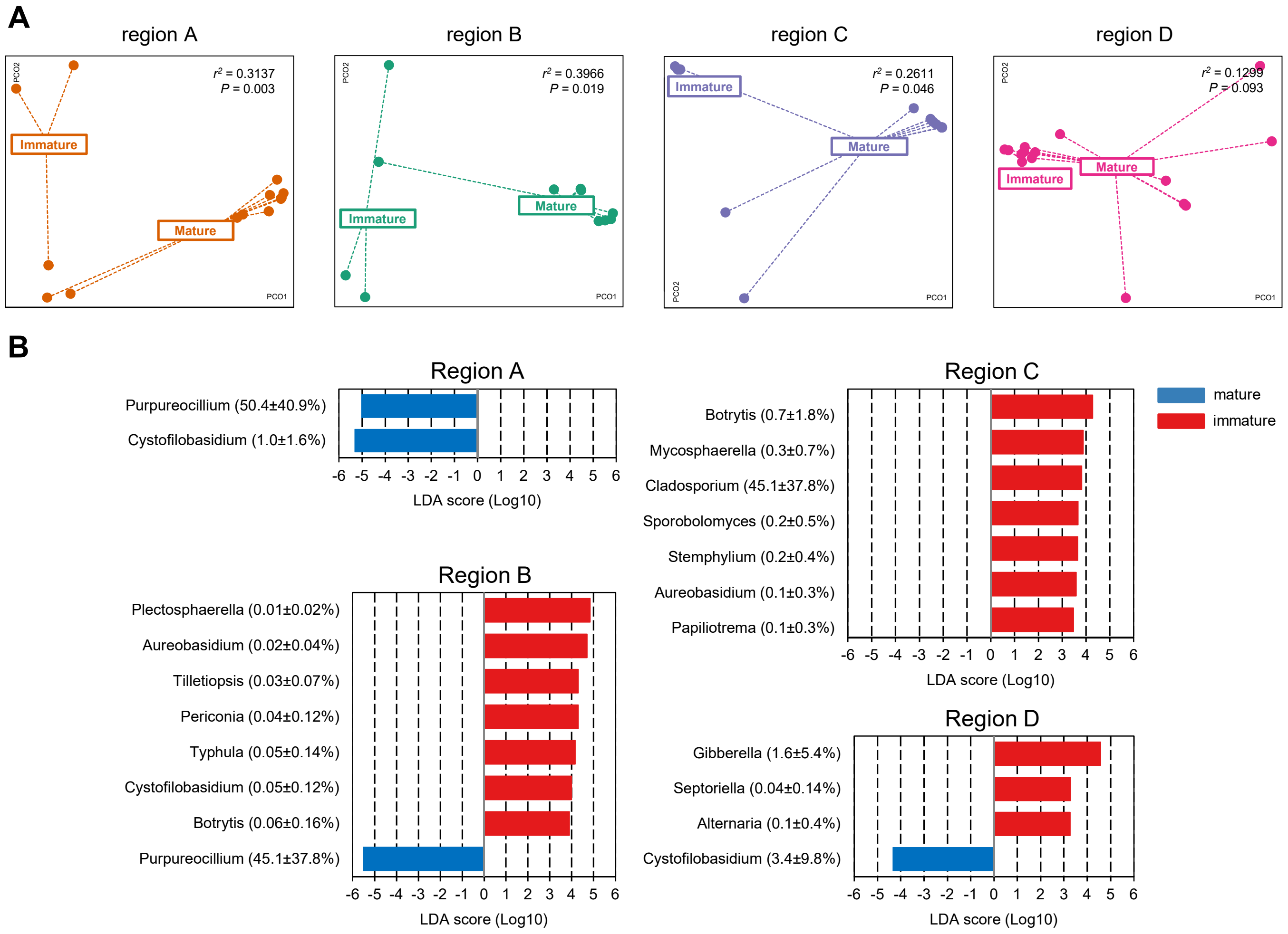

Fig. S4 (A) Immature and mature fungal communities were compared by using Principal coordinates Analysis based on Jaccard distance. (B) Effect size scores of linear discriminant analysis calculated for differences in fungal ASVs abundance between mature and immature samples (logarithmic LDA score >3.0).

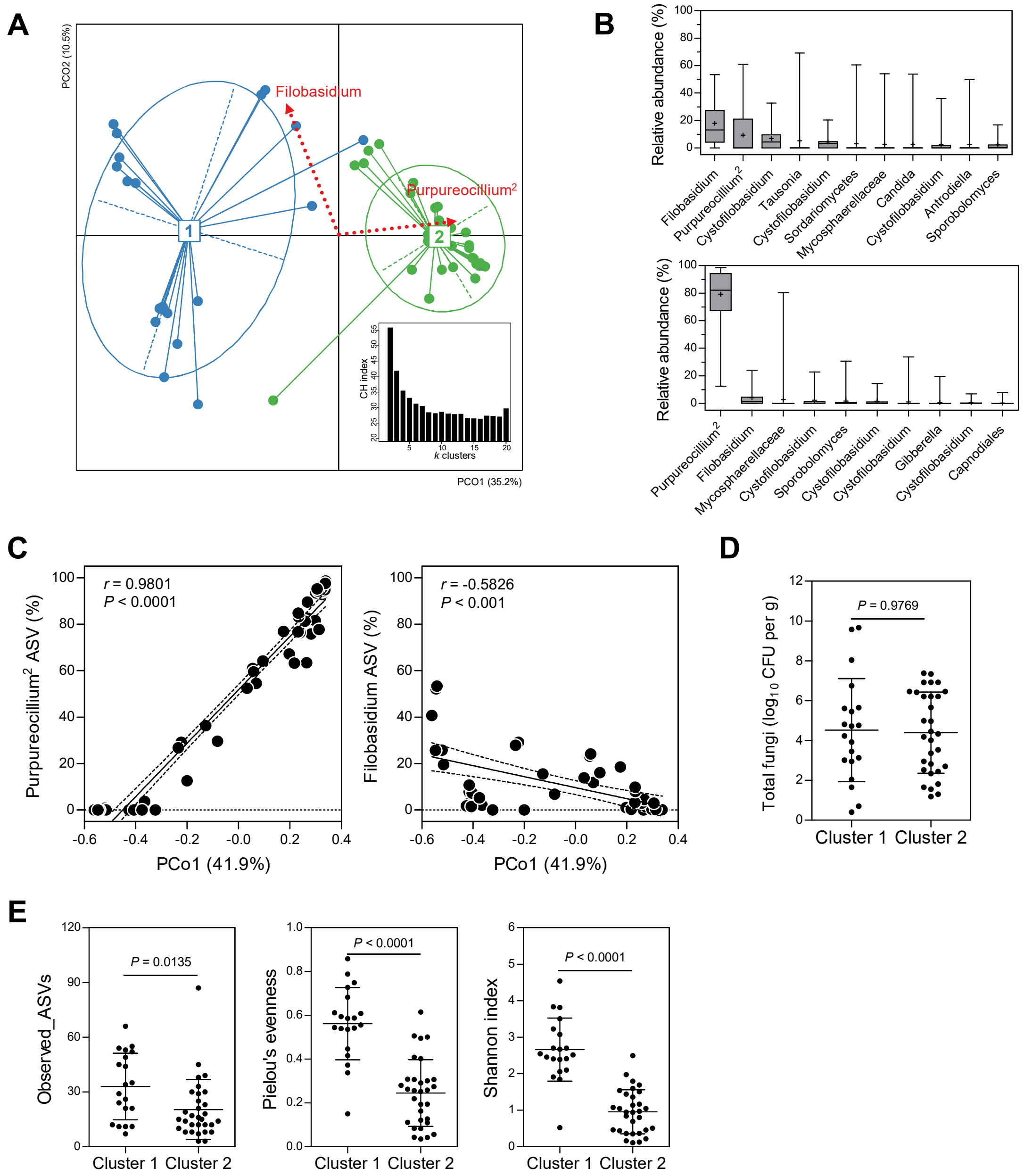

Fig. S5 The clustering of fungal communities of field-grown broccoli. (A) The optimal number of clusters were defined based on Calinski-Harabasz index (lower right panel), and fungal communities were clustered into two groups. Two ASVs that had the strongest correlations with the first axis of PCoA were shown ($r > 0.5$, $P < 0.01$). (B) The abundant ASVs of cluster 1 (upper panel) and 2 (lower panel) types are shown using a rank abundance plot. Box and whisker plots are shown to min and max. (C) The relative abundances of *Purpureocillium* and *Filobasidium* ASVs were correlated with the first axis of unsupervised PCoA plot for Bray-Curtis dissimilarity. Statistical significance was determined by Pearson correlation. Total number of viable fungi (D) and diversity indices (E) of fungal communities of the two clusters were compared. All data are mean ± SD. Statistical significance was determined by two-tailed Mann-Whitney U test.

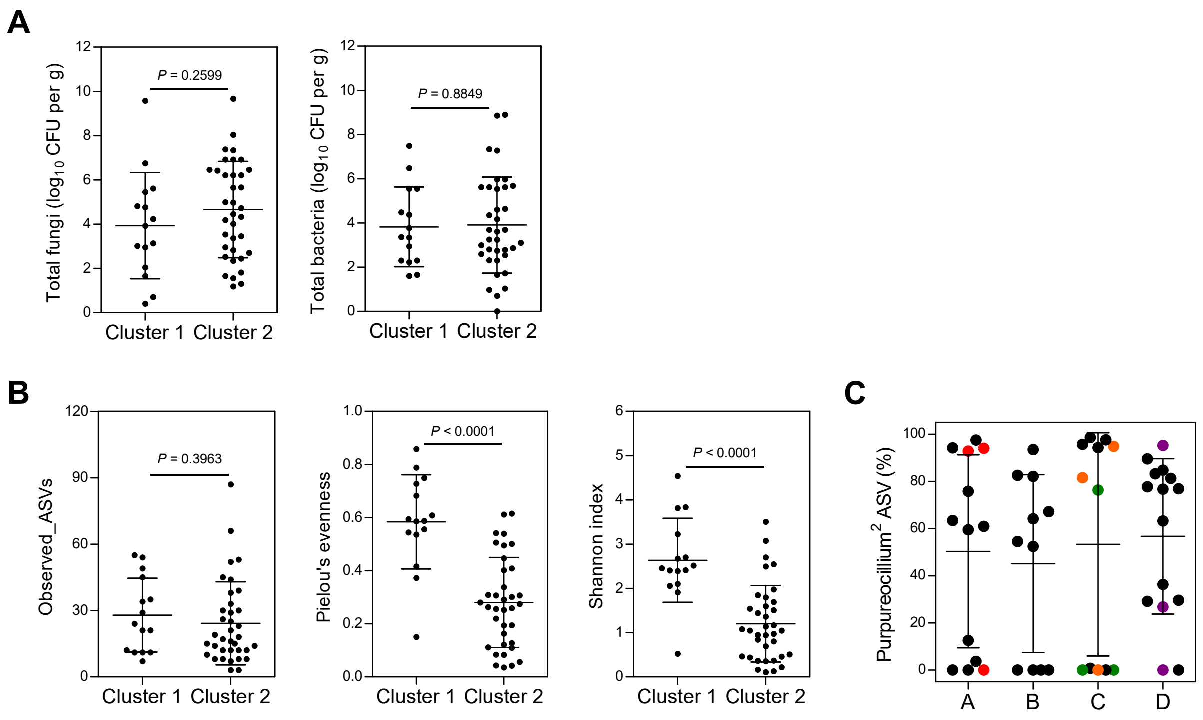

Fig. S6 Total number of viable fungi and bacteria (A), and fungal diversity indices (B) were compared between *Filobasidium* and *Purpureocillium* types. All data are mean ± SD. Statistical significance was determined by two-tailed Mann-Whitney U test. (C) The abundance of the *Purpureocillium* ASV in the communities of four farming regions. Dots colored in red, orange, green, and purple indicate samples originated from the same farm.
