# Supplementary material for: Composition and variability of core phyllosphere fungal mycobiota on field-grown broccoli
Source: Environ Microbiome. 2023 Mar 1;18:15. doi: 10.1186/s40793-023-00474-0 (PMC9976476; doi:10.1186/s40793-023-00474-0)
Supplement: Supplementary file 2 — Additional file 2: Table S1. The information of sampling locations. Table S2. Sequence processing. [file 40793_2023_474_MOESM2_ESM.pdf]

Table S1. The information of sampling locations

Sample ID		Sampling date	Farm ID	Weather station (latitude, longitude)	Distance from weather station (km)	Temperature (°C)[1,2]	Relative humidity (%)[1,2]	Precipitation (mm)[1]	Wind speed (m/s)[1,2]	Insolation (hr)[1]	Soil temperature (°C)[1,2]	Soil moisture (%)[1,2]
A	1, 2, 3	Nov 1st, 2014	A1	33°15'45.6"N 126°16'05.3"E	5.47	17.5	89.8	1.0	2.6	10.0	18.7	38.9
	4, 5, 6	Jan 11th, 2015	A2		4.50	6.5	64.7	0.0	4.4	9.8	6.7	35.8
	7, 8, 9	Jan 11th, 2015	A3		4.41	6.5	64.7	0.0	4.4	9.8	6.7	35.8
	10, 11, 12	Jan 24th, 2015	A4		3.93	5.1	76.6	0.0	1.7	11.9	6.9	40.6
	13, 14, 15	Jan 24th, 2015	A5		4.24	5.1	76.6	0.0	1.7	11.9	6.9	40.6
	16, 17, 18	Jan 24th, 2015	A6		3.96	5.1	76.6	0.0	1.7	11.9	6.9	40.6
B	19, 20, 21	Nov 8th, 2014	B1	33°23'18.9"N 126°17'22.1"E	2.77	11.8	94.4	5.0	0.1	2.1	15.6	27.6
	22, 23, 24	Dec 7th, 2014	B2		2.77	6.0	70.8	0.0	0.5	6.1	9.3	30.8
	25, 26, 27	Feb 8th, 2015	B3		3.55	0.2	70.5	1.0	2.9	7.3	6.6	28.6
	28, 29, 30	Feb 8th, 2015	B4		3.56	0.2	70.5	1.0	2.9	7.3	6.6	28.6
	31, 32, 33	Feb 8th, 2015	B5		3.49	0.2	70.5	1.0	2.9	7.3	6.6	28.6
C	34, 35, 36	Dec 3rd, 2014	C1	33°31'42.7"N 126°37'31.0"E	0.36	6.7	73.6	8.5	1.1	4.6	10.2	40.4
	37, 38, 39	Jan 8th, 2015	C2		0.38	4.8	64.1	0.0	1.6	6.1	7.1	37.4
	40, 41, 42	Jan 22th, 2015	C3		0.89	6.0	76.2	3.0	1.4	5.6	7.7	38.9
	43, 44, 45	Jan 22th, 2015	C4		0.89	6.0	76.2	3.0	1.4	5.6	7.7	38.9
	46, 47, 48	Jan 22th, 2015	C5		1.90	6.0	76.2	3.0	1.4	5.6	7.7	38.9
D	49, 50, 51	Nov 17th, 2014	D1	33°27'24.7"N 126°52'43.1"E	4.25	10.7	74.3	0.0	0.8	6.8	14.0	46.3
	52, 53, 54	Dec 6th, 2014	D2		3.46	2.7	90.0	8.5	0.9	3.4	10.2	53.8
	55, 56, 57	Dec 29th, 2014	D3		4.04	7.1	73.8	0.5	0.7	9.6	8.5	47.6
	58, 59, 60	Dec 29th, 2014	D4		4.07	7.1	73.8	0.5	0.7	9.6	8.5	47.6
	61, 62, 63	Dec 29th, 2014	D5		5.93	7.1	73.8	0.5	0.7	9.6	8.5	47.6
	64, 65, 66	Dec 29th, 2014	D6		5.88	7.1	73.8	0.5	0.7	9.6	8.5	47.6

[1]The climate and soil conditions were measured at the sampling date.

[2]The values indicate averages per sampling day.

Table S2. Sequence processing

Region	Farm ID	Sample ID	#raw reads	#filtered reads	#denoised reads	#non-chimeric reads	rarefied to	#final reads	#ASVs
A	A1	1	50,456	49,099	49,099	48,784	15,000	15,000	25
		2	105,986	46,110	46,110	45,965	15,000	14,998	34
		3	88,801	85,708	85,708	84,658	15,000	15,000	10
	A2	4	61,045	59,472	59,472	58,005	15,000	14,977	61
		5	-	-	-	-	-	-	-
		6	90,265	88,065	88,065	88,054	15,000	14,996	12
	A3	7	71,739	69,960	69,960	69,959	15,000	14,992	17
		8	293,237	91,816	91,816	90,572	15,000	15,000	12
		9	-	-	-	-	-	-	-
	A4	10	26,627	26,142	26,142	24,195	15,000	14,974	62
		11	45,653	44,475	44,475	44,393	15,000	14,977	26
		12	63,528	62,020	62,020	61,751	15,000	14,948	51
	A5	13	-	-	-	-	-	-	-
		14	172,518	168,271	168,271	165,818	15,000	14,972	47
		15	241,139	233,778	233,778	232,508	15,000	14,817	113
	A6	16	51,979	50,794	50,794	49,292	15,000	14,994	107
		17	-	-	-	-	-	-	-
		18	-	-	-	-	-	-	-
B	B1	19	34,107	15,582	15,582	15,357	15,000	15,000	26
		20	80,897	79,499	79,499	78,740	15,000	14,998	39
		21	78,065	76,912	76,912	76,319	15,000	15,000	44
	B2	22	80,505	78,785	78,785	78,537	15,000	14,997	13
		23	79,693	77,954	77,954	77,944	15,000	15,000	19
		24	82,377	73,968	73,968	73,920	15,000	14,996	28
	B3	25	180,474	153,191	153,191	151,263	15,000	14,995	12
		26	78,982	76,827	76,827	76,609	15,000	14,997	20
		27	-	-	-	-	-	-	-
	B4	28	63,806	62,073	62,073	60,652	15,000	14,984	77
		29	-	-	-	-	-	-	-
		30	56,421	54,584	54,584	54,566	15,000	15,000	10
	B5	31	82,677	80,320	80,320	79,982	15,000	14,990	33
		32	-	-	-	-	-	-	-
		33	-	-	-	-	-	-	-
C	C1	34	-	-	-	-	-	-	-
		35	31,257	30,176	30,176	29,206	15,000	15,000	49
		36	62,719	60,711	60,711	59,775	15,000	14,983	60
	C2	37	38,908	37,475	37,475	37,434	15,000	14,997	22
		38	76,996	74,476	74,476	74,476	15,000	14,999	18
		39	86,007	82,957	82,957	82,273	15,000	15,000	17
	C3	40	21,142	20,039	20,039	19,753	15,000	15,000	40
		41	138,206	126,500	126,500	119,523	15,000	14,998	16
		42	61,293	58,947	58,947	58,871	15,000	14,969	54
	C4	43	-	-	-	-	-	-	-
		44	59,554	57,767	57,767	57,767	15,000	14,995	9
		45	52,433	43,799	43,799	43,796	15,000	14,982	24
	C5	46	145,619	140,550	140,550	140,299	15,000	14,986	21
		46	157,418	148,384	148,384	148,332	15,000	14,995	12
		48	-	-	-	-	-	-	-
D	D1	49	245,909	205,926	205,926	198,957	15,000	14,996	20
		50	82,187	79,656	79,656	79,376	15,000	14,988	26
		51	82,863	80,091	80,091	79,984	15,000	15,000	17
	D2	52	104,043	101,678	101,678	101,068	15,000	14,991	26
		53	75,842	73,678	73,678	73,190	15,000	14,999	39
		54	-	-	-	-	-	-	-
	D3	55	-	-	-	-	-	-	-
		56	78,042	76,193	76,193	75,829	15,000	14,994	37
		57	215,475	200,283	200,283	194,910	15,000	14,999	31
	D4	58	-	-	-	-	-	-	-
		59	57,239	55,413	55,413	55,259	15,000	14,990	48
		60	59,164	57,268	57,268	57,259	15,000	14,984	31
	D5	61	176,064	158,065	158,065	157,202	15,000	14,988	19
		62	203,485	198,394	198,394	192,594	15,000	14,949	66
		63	53,121	51,869	51,869	50,902	15,000	14,983	52
	D6	64	57,690	55,498	55,498	55,498	15,000	15,000	8
		65	93,251	90,458	90,458	87,993	15,000	14,974	55
		66	229,594	223,407	223,407	217,201	15,000	14,985	62
